# Spoken Word Recognition Enhancement Due to Preceding Synchronized Beats Compared to Unsynchronized or Unrhythmic Beats

**DOI:** 10.3389/fnins.2017.00415

**Published:** 2017-07-18

**Authors:** Christos Sidiras, Vasiliki Iliadou, Ioannis Nimatoudis, Tobias Reichenbach, Doris-Eva Bamiou

**Affiliations:** ^1^Clinical Psychoacoustics Laboratory, Neuroscience Division, 3rd Psychiatric Department, Aristotle University of Thessaloniki Thessaloniki, Greece; ^2^Department of Bioengineering, Imperial College London London, United Kingdom; ^3^Faculty of Brain Sciences, UCL Ear Institute, University College London London, United Kingdom

**Keywords:** speech processing, auditory processing, hearing, dynamic attention theory, neural oscillations, speech segmentation, rhythm, psychoacoustics

## Abstract

The relation between rhythm and language has been investigated over the last decades, with evidence that these share overlapping perceptual mechanisms emerging from several different strands of research. The dynamic Attention Theory posits that neural entrainment to musical rhythm results in synchronized oscillations in attention, enhancing perception of other events occurring at the same rate. In this study, this prediction was tested in 10 year-old children by means of a psychoacoustic speech recognition in babble paradigm. It was hypothesized that rhythm effects evoked via a short isochronous sequence of beats would provide optimal word recognition in babble when beats and word are in sync. We compared speech recognition in babble performance in the presence of isochronous and in sync vs. non-isochronous or out of sync sequence of beats. Results showed that (a) word recognition was the best when rhythm and word were in sync, and (b) the effect was not uniform across syllables and gender of subjects. Our results suggest that pure tone beats affect speech recognition at early levels of sensory or phonemic processing.

## Introduction

Speech perception may be a more demanding process for children compared to adults, taking into account their lower degree of familiarity regarding novel linguistic information and their developing central auditory nervous system (Kaushanskaya et al., 2013; Levi, 2015). A typically developing child has the same inherent focus on speech as opposed to other auditory stimuli as an adult, but more cognitive and language constraints, as these skills are still developing in childhood. As a child's perception of the environment is based on both existing knowledge and incoming sensory information (Watson et al., 2014), investigating how auditory speech perception may be enhanced is of importance. In addition investigating deviations from normal behavior differentiations in children diagnosed with auditory processing disorders that may impact learning, communication, and academic achievements is essential. This paper assesses whether rhythm may have a positive impact on spoken word recognition in school aged children.

It has been proposed that universally children first learn to process whole sentences before breaking them down into smaller units (Metsala and Walley, 1998), by means of a “temporal scaffolding mechanism” that structures speech input and output in time (Ivry, 1996; Tierney and Kraus, 2016). The brain has to be highly effective in detecting regularities of speech, since speech rhythm is fluctuating. That is to say, a timing monitoring mechanism should be able to identify evolving patterns in time within auditory signals whose regularities are not always easy to detect. This mechanism develops early in life, with native language rhythmic patterns being favored by infants as early as 6 months of age (Nazzi and Ramus, 2003) and with speech stream in children diagnosed segmentation taking place by the age of 8 months. Word learning in childhood is based on selective focus on specific acoustic characteristics of the auditory stimuli, which assists in rapid pattern recognition despite speech complexity (Bergelson and Swingley, 2013). This mechanism may be disrupted in children with central auditory processing disorders and may account for lack of music appreciation and deficits in consonants perception due to temporal processing deficits that have been reported in these populations (Iliadou et al., 2014; Musiek and Chermak, 2014).

Giraud and Poeppel (2012) have proposed a model for the mechanism that segments speech into smaller units, at the level of neuronal function. In their model they suggest that the neuronal oscillations of the auditory cortex are synchronized with the incoming syllables, by means of continuous adjustments to the incoming auditory signal's characteristics. This process results in periodic cortical oscillations that function as a temporal grid, by means of which speech is segmented into small chunks of auditory information at the level of syllables (syllabic parsing) and phonemes. Presence of rhythm affects auditory perception as evidenced by several behavioral and electroencephalography studies and according to the Dynamic Attending Theory (DAT; Jones and Boltz, 1989; Large and Jones, 1999; Bolger et al., 2013; Meltzer et al., 2015). DAT proposes in particular, that perception of rhythm evoked by an external auditory signal results in periodic neural oscillations and oscillations in attention. Hence, stimuli that are aligned with high neuronal excitability are better processed than stimuli that are aligned with low neuronal excitability.

The relation between rhythm and language has been studied for the last several decades. Rhythm is defined as being a psychological plausible pattern that is not relying on division between languages (Arvaniti, 2009). Evidence for the existence of common perceptual mechanisms for both, come from several different lines of research, including psychoacoustic, electroencephalography, and imaging studies. Linguistic studies that have focused on syllabic rates across different languages including Greek, reveal periodicities around 3–5 Hz (200–333 ms; Baltazani, 2007; Tilsen and Johnson, 2008; Wong et al., 2011; Tilsen and Arvaniti, 2013). Studies on the acoustics of speech have found that the dominant component of the amplitude envelope is found in temporal modulations around the same frequency, i.e., in the range of 4–8 Hz (Chi et al., 1999; Chandrasekaran et al., 2009; Elliott and Theunissen, 2009). Auditory cortex functioning reveals focus on temporal characteristics of speech. That is, behavioral, electroencephalography and imaging studies using simple auditory stimuli and speech have shown that the auditory cortex is “tuned” to the syllabic rate, and shows selective sensitivity for frequencies around 4 Hz (Edwards and Chang, 2013; Picton, 2013; Overath et al., 2015). This tuning implies the existence of built-intiming mechanisms that use language syllabic regularities as informational cues to process incoming speech.

Several studies have shown that rhythm perception skills correlate with speech perception and production. Slater and Kraus (2016) assessed speech in noise recognition and the ability to discriminate between pairs of short musical phrases that differed in rhythmic content by means of the Musical Ear Test in musicians and non-musicians (Wallentin et al., 2010). They found that musicians scored better in both tasks compared to non-musicians, and that these two measures correlated across both groups, suggesting that a common mechanism is required for both tasks performance. The relation between rhythm perception and reading ability has also been studied (Grube et al., 2012; Goswami et al., 2013; Bekius et al., 2016). Grube et al. (2012) measured reading/phonological skills and perceptual rhythm skills through psychoacoustic testing in typically developing 11 year-old children. They found significant correlations between the language and rhythm tests, consistent with the findings by Grube et al. (2013) and Bekius et al. (2016). Goswami et al. (2013) found a severe deficit of musical beat patterns perception in children with dyslexia as opposed to typically developing ones and linked this deficit with linguistic processing. Rhythm skills were found to be highly correlated with grammar skills (Gordon et al., 2014) and phonological awareness (Moritz et al., 2013) substantiating the neurocognitive overlap between music and language processing.

This paper focuses on how rhythm may have a positive impact on spoken word recognition in school aged children. The aim of this study was to assess the effect of rhythm induced by isochronous beat sequences on word in babble recognition scores. A new test, the Word Recognition—Rhythm Component (WRRC) was developed for this purpose. Three separate beat sequences were created and used, (i) a rhythmic sequence in synchrony with the following bisyllabic word, (ii) a non-rhythm beat sequence that served as a baseline performance indicator for the purposes of this study, and (iii) an isochronous but unsynchronized sequence that was used in order to investigate the importance of synchronicity. Baseline performance in this study is defined as the performance when cuing of equal duration (compared to RH condition) is present, while rhythm is lacking. This is a pilot study within a larger study on temporal processing in children diagnosed with Auditory Processing Disorder. The aim was to test the hypothesis that spoken word recognition would be best when words and beats where in sync in typically developing school-aged children. The next step will be to implement the WRRC test for each child in a closed field approach through headphones in a sound-treated booth and compare with the Greek Speech in Babble test (SinB) baseline results to determine if spoken word recognition improves due to preceding rhythmic beats. In this specific open set experiment we developed three conditions. The first one was a rhythmic sequence in sync with the following bisyllabic word, that was used to test the rhythm effect on spoken word recognition. The second one was a non-rhythm beat sequence preceding the bisyllabic word, that was used as a baseline and in order to test the possibility that the preceding auditory stimuli would raise awareness of the incoming speech sounds in which case we would obtain a similar perceptual score as in the rhythm condition. The third condition was an isochronous but unsynchronized sequence used in order to investigate any differences between the condition of beats and words being in sync with the condition of the two different auditory stimuli being out of sync. Sex differences were also examined, as boys are reported to have higher incidence of auditory processing perceptual deficits as well as neurodevelopmental disorders (e.g., dyslexia, El Sheikh et al., 2016; learning disability, Fortes et al., 2016).

Based on the reported relations between language, attention, and rhythm, our hypothesis was that the existence of preceding rhythmic beats in synchrony with the words would enhance the word recognition in babble. According to the DAT theory in particular, it is expected that sensory responses to stimuli that are aligned with the high excitability phase of the oscillation are amplified, while the exact opposite would be true for sensory responses to stimuli that are aligned with the low excitability phase of the oscillations. It would thus be expected that recognition of syllables that coincide with the high excitability phase will be enhanced. Effects of differencial processing in the presence of rhythm explained in terms of DAT include visual processing (Escoffier and Tillmann, 2008; Escoffier et al., 2010; Bolger et al., 2013, 2014; Miller et al., 2013) auditory processing (Bolger et al., 2013, 2014) and linguistic-semantic processing (Poulin-Charronnat et al., 2005).

## Methods

### Participants

Primary school children of the 5th year were included in the study. Two classes with a total of 27 10 year-old children (age range 10 years and 1 month to 10 years and 11 months) were evaluated. The inclusion criteria were age appropriate writing skills (according to the teachers' report), normal hearing thresholds (based on teachers' report) and Greek as first language. A total of 26 children (10 males, 16 females) were recruited for analysis (one child was excluded due to the presence of writing deficits). This study was carried out in accordance with the recommendations of the Greek Ministry of Education (Ref. no. F15/1965/14505/D1) with written informed consent from the parents/legal guardians of all subjects. This is in accordance with the Declaration of Helsinki. The study was approved by the Ethics and Bioethics Committee of the Aristotle University of Thessaloniki.

### Auditory stimuli

A fifty word list (List 3 in Iliadou et al., 2006) that consisted of natural spoken Greek bisyllabic words was used to create 3 new lists for the purposes of this study. The words were recorded in a sound-treated booth by a female individual with a sampling rate of 22,050 kHz. Issues taken under consideration for the development of List 3 included: the use of the shortest possible words to minimize redundancy; frequency of words use relative to the frequency of occurrence of Modern Greek phonemes; the frequency of relative stress patterns; and the distribution of vowels in the stressed syllable. List equivalence in terms of phoneme balance and stress pattern balance was determined in quiet (Iliadou et al., 2006). Subsequently these lists were used to test children for the diagnosis of Auditory Processing Disorder (Sidiras et al., 2016) and were found to be both appropriate and effective for pediatric use. Each of the three new lists consisted of 11 words (68.75%) stressed on the first syllable and 5 (31.25%) stressed on the second syllable. These percentages are very close to the ones present in the development of word lists (70% first syllable stressed and 30% second syllable stressed) by Iliadou et al. (2006) that reflect the percentages present in bisyllabic words in modern Greek language. These words were otherwise randomly chosen for each list and two words (both stressed on the first syllable) from the original list were discarded.

**Figure 1:**
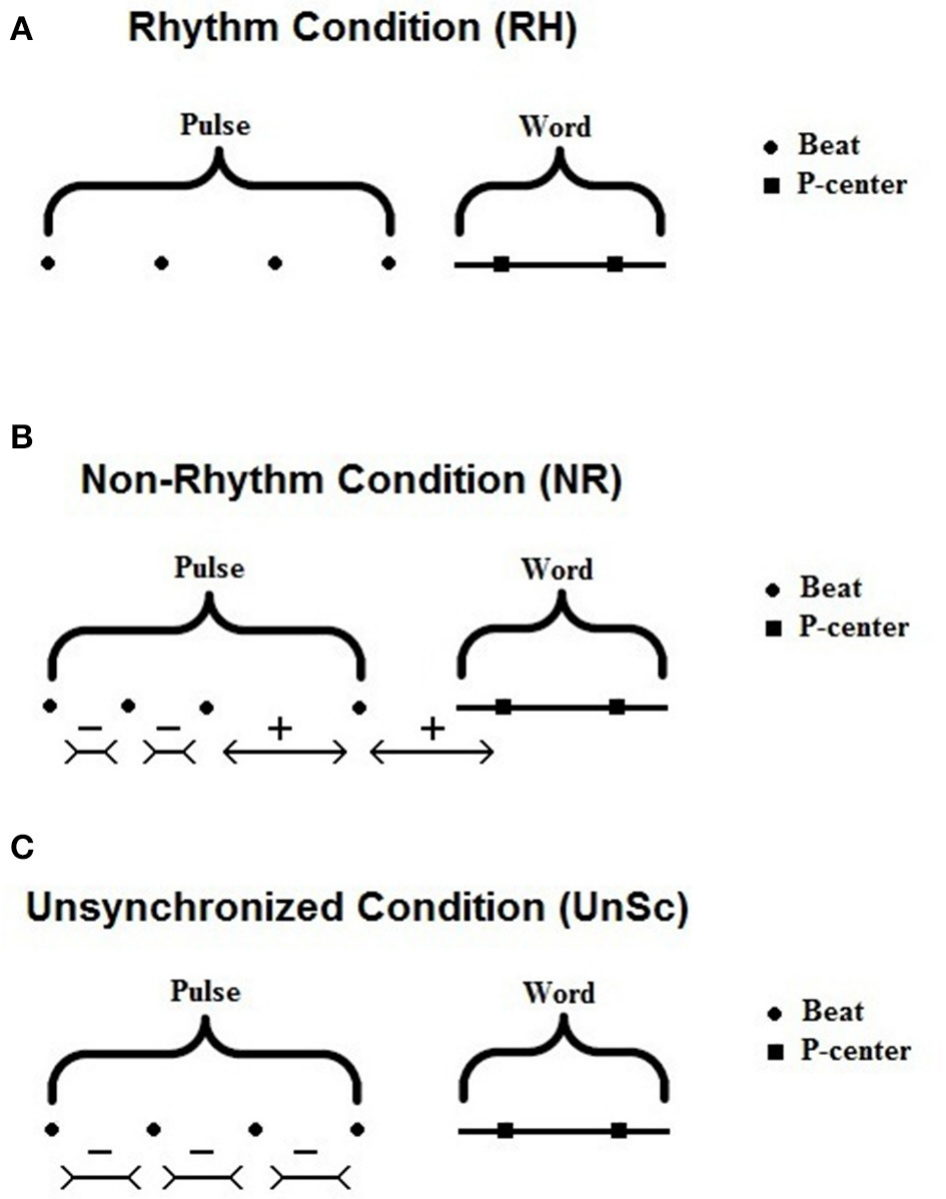
Illustration of **(A)** Rhythm (RH), **(B)** non-Rhythm (NR), and **(C)** Unsychronized Condition (UnSc). In RH condition, beats and P-centers are equidistant. In NR condition, distances between beats and P-centers are distorted by either shortening or lengthening by 30% (type A is shown here; see Table 1). In UnSc condition, IBIs (inter-beat intervals) are shortened by 10%. Beats are equidistant but P-centers do not follow the beats.

Auditory stimuli were presented in three conditions, Rhythm Condition (RH), non-Rhythm Condition (NR), and Unsynchronized Condition (UnSc) in a randomized order. In all conditions, the stimuli consisted of a preceding brief 4 beat sequence (1,000 Hz, 15 ms) and a word in babble. This sequence was adjusted to words based on syllables' Perceptual centers (P-centers), and the adjustment was different for each condition (see Figure 1 and Section “WRRC Conditions”). The P-center of a syllable is defined as “its psychological moment of occurrence” (Morton et al., 1976). We used musicians to measure the words P-centers, as musicians have more finely tuned temporal processing skills than non-musical subjects (Gaab et al., 2005) and in order for this study to have a measurable index for the auditory psychoacoustic perception of rhythm. The background multitalker babble used was recorded at the university student cafeteria using a highly sensitive microphone (Shure SM 58) routed directly to a personal computer with Cool Edit software during rush hour (Sidiras et al., 2016). In all conditions, the babble was inserted within the stimulus starting 1.5 inter-P-centers intervals (IPI) before the 1st P-center. The envelope of the babble followed a linear ramp (fade in) of 1 IPI duration, (ramp ending at 0.5 IPIs before the 1st P-center), was then kept constant and ended without A ramp 2–3 hundreds of milliseconds after the word.

### P-centers determination

P-centers' determination was executed on Matlab software (version R2011a), at a sampling rate of 44,100 Hz. First, a low-accuracy estimation was achieved by visual inspection of the word's envelope, placing 2 *P*-values (estimated P-centers), one for each syllable, respectively, at local maxima. It should be stressed that visual inspection was used only as a starting point. Two professional musicians (CS and SE) with 15 years of musical experience were recruited for determination of P-centers. CS is a string instrument player, and a graduate of Aristotle University, School of Music studies, and SE is a string and percussion instruments player, and a graduate of University of Macedonia, School of Science and Art.

The following procedure was repeated for each word, and was run for each musician separately. First, a stimulus was generated in the following way. A 4-beat sequence (15 ms and 1,000 Hz) was generated in Matlab, whose inter-beat internal (IBI) matched the word's IPI. Then, the word was added to the stimulus, placed a way that if the sequence did not stop, 5th and 6th beats would co-occur with the word's 1st and 2nd visually estimated *P*-values, respectively. Both musicians regarded visual estimation as sufficient in that sequence and words were perceived as “being more or less as part of the same rhythm.”

During the process, the word was presented as a whole, not as separated syllables. The first syllable's *P*-value was adjusted first. The sequence and word were presented via headphones (Sennheizer HD 380pro). And after each presentation, the operator adjusted in a staircase fashion the *P*-value in steps of 1,000 samples (corresponding to 22.7 ms) according to musician's instructions. The possible responses were (a) “syllable seems to be delayed,” (b) “syllable seems to come too early,” and (c) “it sounds good”. In the case of CS the response was silent, since he was also the software operator, for SE the response was oral. If the response for the first presentation was (a), *P*-value was increased, until the response became (b) for 2 consecutive presentations. Then *P*-value was decreased until the response became (a) for 2 consecutive presentations. If the first response was (b), the initial direction of *P*-value was the opposite (decreasing), and if the first response was (c), a quasi-random choice was made by the operator. This process was repeated 2 times, i.e., 4 runs (presentations whose direction of *P*-values' change was the same). The average of the two border values from the 2 last runs within which the response was “it sounds good” was used for fine adjustments in steps of 500 samples (corresponding to 12.2 ms) in the exact same way. In this step of the procedure the musician was instructed to “pay more attention for fine adjustments,” and presentation often were repeated without response, as the musician required a replay. The average, which was calculated in the same way as mentioned above, was considered as musician's final estimation. The same procedure was repeated for the 2nd syllable.

When an adjustment was made to P-center timing in the staircase paradigm, this immediately affected IBI (Inter-Beat-Interval) of the preceding tones as well as the distance between the last tone and the first P-center. IBI was always equal to IPI. During the 1st syllable P-center staircase estimation, *P-*value for the second syllable was fixed, according to visual inspection as described above. Hence, adjusting the *P*-value for the 1st one affected the Inter-P-centers'-Interval (IPI) and consequently the IBI. The same was true during the 2nd syllable P-center adjustment. However, these changes were quite small, equal to the step size, i.e., 22.7 and 11.9 ms for the 1,000- and 500-step sessions, respectively. For each word, the total range of change (across all trials) did not exceed 90 and 45 ms for the 1,000 and 500 step sessions, respectively. Spacing between the last beat and the onset of the first syllable was not fixed across trials. These changes were also rather small, equal to the step size, that is 22.7 and 11.9 ms for the first (step 1,000) and second session (step 500), respectively. For each word, the total range of change (across all trials) did not exceed 90 and 45 ms for the 1,000 and 500-step sessions, respectively.

The whole procedure lasted about 3 h in total for each musician, and was complete in two sessions of equal duration. This methodology allowed the estimation of P-centers with accuracy in the order of 1–2 tens of milliseconds, as seen by the magnitude of differences between musicians' estimations (mean = 17 ms, *SD* = 14 ms). Mean IPI was equal to 329 ms (~3 Hz), *SD* = 55 ms, min = 224 ms, max = 434 ms, skewness and kurtosis equal to −0.041 and 519, respectively.

### WRRC conditions

In all three conditions, the preceding sequence consisted of 4 beats. Care was taken so that the stimuli's total duration was not affected by condition. The beat was a brief pure tone (15 ms duration, 1,000 Hz). Supplementary Audio samples of stimuli are also available online.

In RH the preceding sequence is isochronous and IBI are equal to IPI of each word. Note that IPI are not constant across words, hence each word is preceded by a different kind of sequence in terms of IBI. The word is placed in a way that the interval between the 4th beat and 1st P-center is equal to the IBI, hence both beats and P-centers are parts of an isochronous pulse (Figure 1). Due to this, P-centers are perceived as part of the rhythm induced by the preceding sequence, as they would co-occur with the beat of the sequence, if the sequence was to continue beyond the 4th beat. This produces a sensation of the word being synchronized with the sequence.

In NR, the sequence and the word are arranged is the same way as in RH, but the first 4 intervals (3 between beats and 1 between last beat and 1st P-center) are distorted in a way that the total rhythm is not perceived as isochronous. That is, intervals were distorted by either lengthening or shortening by 30% (Madison and Merker, 2002) in one of the 6 types shown in Table 1. Note that all types were designed in a way that the presence of duration distortion does not affect the total duration of the 4 intervals. Type of each NR presentation was chosen in an ordinal manner, i.e., first NR presentation—type A, second one—type B etc. Distorting inter-beat-intervals by a factor of 30% in a random fashion results in a sequence that is no more perceived as having rhythm (Madison and Merker, 2002).

**Table 1:** Types of distortion of NR condition.

**Type**	**1st interval**	**2nd interval**	**3rd interval**	**4th interval**
A	−	−	+	+
B	−	+	−	+
C	−	+	+	−
D	+	−	−	+
E	+	−	+	−
F	+	+	−	−

*“−” stands for shortened interval and “+” for lengthened interval*.

Last, in UnSc, the pulse and the word is arranged in the same way as in RH, but all IBI was shortened by 10%. Word position did not change, hence total duration was kept the same. This way, the pulse did induce a rhythm but this rhythm is not preserved by the following word. That is, P-centers are not perceived as part of the rhythm induced by the preceding sequence, as they would not co-occur with any beat beyond the sequence, if it was to continue. Slower IBIs were not used in UnSc condition for 2 reasons: The first reason has to do with brevity, as an extra condition would result in longer testing duration. The second reason had to do with technical issues. Words' position was fixed such that 1 P-center was at 4 IPI, since we had already decided that the condition employed should not affect stimuli's total duration (see above). If IPI was increased, the last beat would get very close to the word. In that case, in some instances in which the1st P-center occurred much later after the syllable's onset, the fourth beat might even occur after the onset of the 1st syllable.

It should be mentioned that distance between last beat and word's onset was affected by condition, and in the case of NR condition, by the type of distortion. Technical issues arises when trying to reconcile, since stimuli's duration is affected by this distance. We opted to keep stimuli's duration unaffected, and acknowledge this as a confounding factor.

### Procedure

The WRRC test was delivered through a laptop and open field speakers (TurboX D-400 2.1, 40 Watt, Frequency Response 35–20,000 Hz) that were placed in the center of each class facing the children. The WRRC was presented at 60 dBA (at 1 m), in two experimental trials, one for each class. Signal-to-Babble Ratio was set to 1.3 dB. Children were seated 1.5 m away from the speaker, in a semi-circular arrangement, ensuring that the distance between each child and the speaker (situated at the center of the circle) was the same. Each child was given a sheet with 48 cells, one for each word. Children were instructed to write down each word they listened to and not to worry about correct spelling. The experimenter (an experienced teacher, the first author) ensured that each word was delivered after all children were done writing the previous-one. Care was also taken that children did not cheat. For each word, 1 point was given for each correct syllable recognition. If the whole word was recognized correctly, 2 points were given.

Regarding subjects' scores, the test gave 3 primary output measures, RH, NR, and UnSc scores. These scores correspond to the total points (correct syllables) that were given for each condition, respectively. Given that 16 bi-syllabic words were presented for each condition, the maximum possible score for each condition was 32. Six secondary output measures were also given from the test, RH1, RH2, NR1, NR2, UnSc1, and UnSc2, which correspond to the condition and the score only for first and second syllable, respectively. For example, RH1 is the number of the first syllables of each word that were recognized correctly for condition RH, while RH2 refers to the same condition for the second syllables that were recognized. Note that for all secondary measures, maximum possible is equal to the total number of words, i.e., 16.

### Classroom acoustics

Background noise level was measured in each classroom via a sound level meter (Dr. Meter MS10). Three different measurements were implemented for each classroom. Mean noise level was at 35 dBA for each classroom. Both classes were located at the upper floor of a two-stories building, occupying 13 class-rooms as a total. Classrooms' size was 8.5 m length, 4 m width, and 3 m height, and 6 m length, 4.5 m width, and 3 m height, respectively. In the first class there were two windows of size 4, 1.7 m and 1, 1.3 m, respectively, located in opposite walls. In the second class there was one window of size 4, 2 m. In both classes there was no carpet. Schools' teachers (one of the authors CS included) reported that both classrooms' reverberation was quite low, such that it did not had a negative effect on speech intelligibility. As optimal speech intelligibility has a strict upper limit of 0.4–0.5 s of reverberation time (Bradley, 1986) and this is known to be present in classrooms having a volume of 200 m^3^ (Picard and Bradley, 2001), neither of the two classrooms of the present experiment reached the upper limit of reverberation time. Thus, perceived intelligibility of the two classrooms as a factor of reverberation was not expected to differ. Testing took place with-in school schedule, between 10 and 11 o'clock.

### Statistical analysis

Results did not follow a normal distribution under the criterion of skewness and kurtosis *z-*values ranging between −1.96 and 1.96 (Cramer and Howitt, 2004; Doane and Seward, 2011). Non parametric tests were used for statistical analysis, i.e., Kruskal–Wallis, Mann–Whitney, Spearman correlation analysis, and Friedman's test. Subsequently Kruskal–Wallis analyses between all possible combinations of groups were executed whenever three groups were compared in the original analysis. In this case, alpha levels were set to 0.017 and 0.003 under Bonferroni correction, instead of the typical levels of 0.05 and 0.01. The same alpha levels were also used in correlation analysis, were correlations between three groups were assessed. Whenever differences between small sample sizes was assessed, Mann–Whitney test was preferred instead of Kruskal–Wallis, as the former has been tested for its validity, although it is a more conservative test (Fagerland and Sandvik, 2009). Effects that were examined through statistical analysis were: (a) effects of condition on subjects' performance, (b) correlation between condition scores, (c) effects of sex on condition and interaction between sex and condition. Effects of condition on performance were investigated through within-subjects Friedman's test. Since Kruskal–Wallis analysis between three groups do not indicate which groups differ, but only whether there is some difference among the groups, *post-hoc* analysis under Bonferroni correction was executed in order to examine which pairs of groups differ.

## Results

### Effects of condition on listeners performance

**Figure 2:**
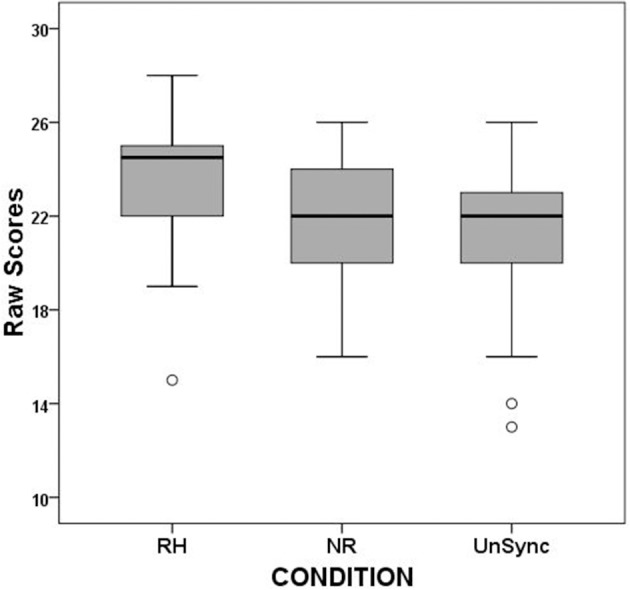
Boxplots of RH, NR, and UnSc scores for all subjects and syllables.

Descriptive statistics (median, min, max) and boxplots of RH, NR, and UnSc scores are shown in Table 2 and Figure 2. Friedman analysis was executed to assess effects of condition on listeners' scores. Variables inserted into the analysis were RH, NR, and UnSc scores. The analysis revealed significant differences between RH, NR, and UnSc scores [$\chi_{\left(2\right)}^{2}$ = 13.271, *p* = 0.001]. *Post-hoc* analysis, showed that RH scores were higher (better) than both NR and UnSc scores (*p* = 0.009 and *p* = 0.001, respectively), but no significant difference was revealed between NR and UnSc scores (*p* = 0.835).

**Table 2:** Result scores for each condition are presented for male (*N* = 10), female subjects (*N* = 16) as well as for the total group (*N* = 26).

	**RH**	**RH1**	**RH2**	**NR**	**NR1**	**NR2**	**UnSc**	**UnSc1**	**UnSc2**
Males	**22.5 (3.6)**	13 (1.8)	10 (2.2)	**22 (2.5)**	12 (1.3)	10 (1.5)	**22.5 (4.5)**	12 (2.1)	9.5 (2.7)
Females	**25 (2.4)**	13 (0.7)	11.5 (1.9)	**22 (2.6)**	11.5 (1.3)	10.5 (1.7)	**22 (2.1)**	12 (1.4)	9 (1.1)
Total	**24.5 (3.1)**	13 (1.2)	11 (2.2)	**22 (2.5)**	12 (1.3)	10 (1.7)	**22 (3.2)**	12 (1.6)	9 (1.9)

*Median scores of the number of correctly identifies syllables as well as standard deviation in parenthesis are presented. For each condition, bold scores refer to whole word (perfect score 32), non-bolded scores refer to each syllable score separately (perfect score 16). RH, NR, and UnSc scores refer to words. RH1, RH2, NR1, NR2, UnSc1, and UnSc2, scores refer to syllables; 1 being the first syllable and 2 the second syllable*.

**Figure 3:**
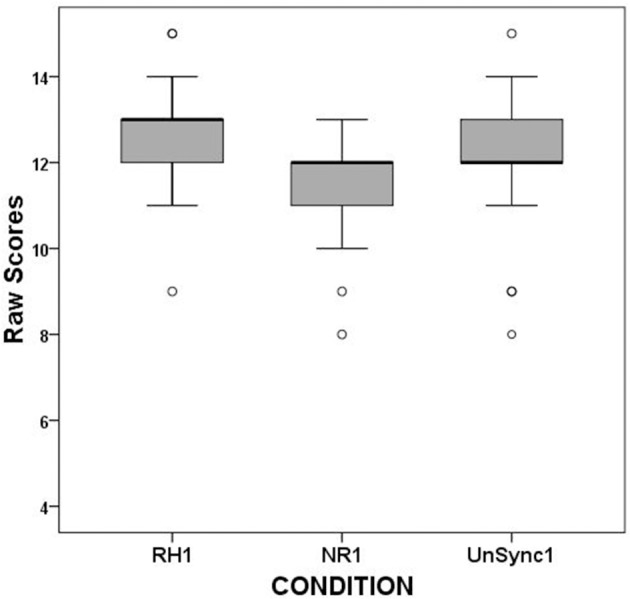
Boxplots of RH1, NR1, and UnSc1 scores for the first syllable of all subjects.

**Figure 4:**
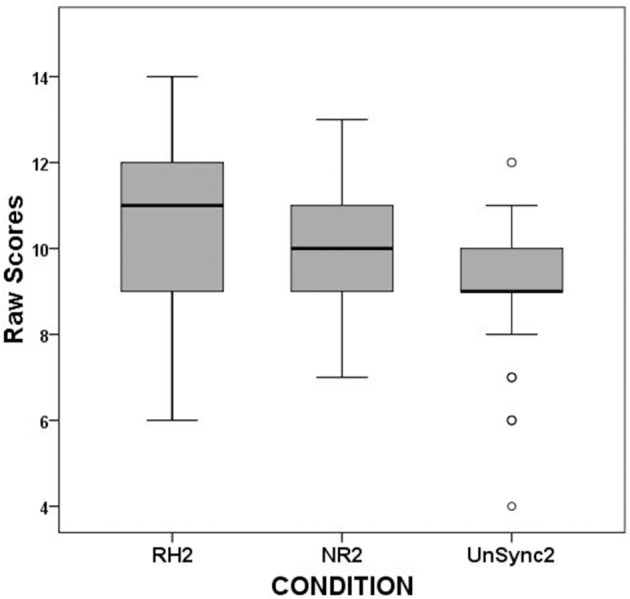
Boxplots of RH2, NR2, and UnSc2 scores for the second syllable of all subjects.

When the same analysis was executed for separate syllables' scores (RH1 vs. NR1 vs. UnSc1, and RH2 vs. NR2 vs. UnSc2, respectively), the analysis yielded significant results for both even if the effect size was larger for the first than the second [$\chi_{\left(2\right)}^{2}$ = 14.0, *p* = 0.001; $\chi_{\left(2\right)}^{2}$ = 7.708, *p* = 0.021, respectively]. *Post-hoc* analysis on first syllable scores revealed that RH1 scores were higher (better) than both NR1 and UnSc1 (χ^2^ = 8.909, *p* = 0.003; χ^2^ = 6.0, *p* = 0.014) and UnSc1 scores was higher than NR1 (χ^2^ = 5.762, *p* = 0.016) (Figure 3). *Post-hoc* analysis on second syllable scores revealed that RH2 scores were higher than UnSc2 (χ^2^ = 6.545, *p* = 0.011), while both differences between RH2 vs. NR2 and NR2 vs. UnSc2 scores were not significant (*p* = 0.180 and *p* = 0.127, respectively) (Figure 4). The effect of rhythm was also investigated separately for stressed and unstressed syllables. The rhythm effect was significantly larger for the 1st unstressed syllables vs. the 2nd unstressed ones (*F* = 6.175, *p* = 0.020). For stressed syllables however, the effect was uniform across syllables (*F* = 0.484, *p* > 0.05).

### Correlation between conditions

Correlation analysis between condition scores was also executed. The only combination that correlated was RH vs. UnSc scores (*r*_*s*_ = 0.438, *p* = 0.025), while correlation between other combination was not significant (RH vs. NR scores: *r*_*s*_ = 0.191, *p* = 0.351; NR vs. UnSc: *r*_*s*_ = 0.094, *p* = 0.647). When the same analysis was executed for separate syllables, RH1 did not correlate with UnSc1 (*r*_*s*_ = 0.142, *p* = 0.488) but RH2 correlated with UnSc2 (*r*_*s*_ = 0.437, *p* = 0.026). Hence, RH vs. UnSc share a total of 19.2% variance, and RH2 vs. UnSc2 19.1%, leaving 80.8%, and 81.9%, respectively unexplained. However, neither of the observed correlations (RH vs. UnSc and RH2 vs. UnSc2) remained significant under Bonferroni correction (alpha level being set to 0.017).

### Effects of sex on condition scores

Differences between boys' (*n* = 10) and girls' (*n* = 16) scores were also investigated. The Mann–Whitney test revealed significant differences only for RH2 scores (*U* = 675.0, *p* = 0.028), with girls scoring higher (better) than boys, while for all other scores no significant differences were present.

## Discussion

To our knowledge, this is the first study that measured effects of beat sequences on words in babble perception in 10 year-old children. When beat sequence rhythm and words were synchronized, a positive effect was observed. That is, isochronous beats that were in sync with words' P-centers (RH condition), enhanced speech in babble recognition compared to when beats were not isochronous (NR condition), but the same was not true when beats and P-centers were not synchronized (UnSc condition). However, we acknowledge that differences in distance between last beat and word between conditions (see Section WRRC Conditions) may play a minor role in these results. These findings are in accordance and expand previous studies of rhythm in language perception for sentences (Przybylski et al., 2013; Kotz and Gunter, 2015) and words (Quene and Port, 2005; Cason and Schon, 2012). This pilot study includes a limitation of using a non-rhythm beat sequence (rather than silence) to serve as a baseline performance indicator for the study purposes. This methodology does not allow to assess whether rhythm does enhance word recognition in babble compared to normal hearing conditions, where no priming exists at all. NR condition's sequence may function as a distractor, that is to decrease word recognition compared to silence^1^. This limitation will be addressed at a later stage through comparison with the SinB test as part of an ongoing PhD thesis. The SinB (Sidiras et al., 2016) is a test showing a clear difference between typically developing children and children diagnosed with Central Auditory Processing Disorder aged 4–13 years old.

Difference between conditions is also suggested by the low correlation between them. RH and NR conditions did not correlate significantly at all. This suggests that these two conditions measure different aspects of speech processing, and that the way priming's rhythm characteristics affects speech processing is not uniform across listeners (see also Henry and Obleser, 2012). If no effect was present, large correlation would be expected. However, some similarity between RH and UnSc condition may exist, as they share a total of 19.2% variance.

In their study, Przybylski et al. measured the effect of listening to regular sequences vs. irregular sequences before sentences on grammatical judgments of children with SLI (Specific Language Impairment), dyslexia, and a matched age control group. They found that for all three groups, judgments were better following regular sequences than following irregular ones. This rhythm effect on speech recognition was present in both typically developing children and those with neurodevelopmental disorders. Kotz and Gunter (2015) measured P600 and N400 responses to incorrect syntactic and semantic information, respectively in sentences, in a patient with Idiopathic Parkinson's disease. Speech presentation took place in three conditions, that is, (a) in absence of rhythm stimulation, (b) after presentation of a 3 min long march music (meter of 4/4, i.e., strong-weak-strong-weak accentuation pattern), and (c) after a 3 min long waltz (meter of 3/4 i.e., strong-weak-weak accentuation pattern) music. The March music rhythm was more relevant to the accentuation pattern in the German patient's language compared to waltz, since the German language involves a strong—weak alteration of accents. As the P600 and N400 results indicated, the patient benefited by the presentation of march music, but not by the waltz music, compared to baseline listening with no rhythm stimulation.

Both the aforementioned and the present study results suggest that the listeners' speech processing benefits by rhythm stimulation when the rhythm of the music or beat sequence fits the speech rhythm, at word as well as at sentence level. However, the observed effects may concern different levels of processing. In Przybylski's et al. and Kotz's and Gunter's studies, the effect of rhythm concerned cognitive language processing (syntactic and semantic processing), while in this study, the effect observed concerned a lower level of processing, i.e., word recognition, with different effects observed for different syllables. We propose that the word effect is due to auditory sensory or phonemic processing enhancement, rather than language related mechanisms, for two reasons. Firstly, sex differences were observed for the 2nd syllable, but not for the first one. If cognitive factors were engaged, we argue that a uniform effect over syllables would be expected. Secondly, perception of words in babble is less prone to cognitive language related processing, compared to sentences (McArdle et al., 2005; McArdle and Hnath-Chisolm, 2009).

Quene and Port (2005) and Cason and Schon (2012) also found effects of rhythm on speech processing. In both studies speech processing was assessed indirectly through a phoneme detection task, and by measuring reaction times, which were lower when rhythm and target stimuli was in sync. Cason and Schon found larger amplitudes for N100 and longer latencies for P300 responses when metrical mismatches occurred between rhythm and target stimuli.

### Rhythm effect and dynamic attention theory

Isochronous beats are known to evoke fluctuations of attention in the form of neural oscillations in auditory cortex (Lakatos et al., 2013; Andreou et al., 2015). Processing of stimuli that are in sync with the peaks of these oscillations is enhanced, while the opposite is true for stimuli that aren't in sync. Our findings may be in line with the Dynamic Attention Theory (DAT), as syllables that were in sync with beats (i.e., RH condition) were better recognized than (a) syllables that were not in sync (i.e., UnSc condition), and (b) syllables for which no rhythm, and hence no oscillations were present (NR condition).

This effect was stronger for the first syllable than for the second one. This difference may be explained when taking into account that a sequence of identical isochronous beats are perceived as a sequence of alternating strong and weak beats (Brochard et al., 2003; Abecasis et al., 2005; Potter et al., 2009). These alternating beats create the perception of a metrical structure (Lerdahl and Jackendoff, 1983; Povel and Essens, 1985), i.e., a subjective alteration of strong and weak accents. The DAT states that perception of metrical structure, i.e., periodic alteration of strong and weak accents, is the result of dynamic fluctuations in attentional resources, peaking at metrically strong positions (i.e., strong accents). Once a sequence of alternating accents is initiated, a perception of rhythm is engaged, and the perception of future events is affected by these accents (Lerdahl and Jackendoff, 1983; Povel and Essens, 1985). In the case of the words that were synchronized with the preceding sequence, the first and the second syllables coincide with the perceived strong and weak accents, respectively. Note that these accents are perceived only because of induction of a rhythm percept, since they do not exist in the actual physical signal and they are distinct from linguistic stress. As a result, more attentional resources are occupied during the first syllables than the second-ones, hence the rhythm effect on word recognition is better for the first unstressed syllables, with uniform rhythm effects for stressed syllables.

### Rhythm effect and speech segmentation model

Our findings can also be interpreted with regards to Giraud's and Poeppel's (2012) speech segmentation model. The rhythm of isochronous sequences activates internal clocks in the brain (Povel and Essens, 1985), before the word is presented. Hence, a temporal grid, according to which speech segmentation may take place (Giraud and Poeppel, 2012), is already at work when the incoming word in noise signal arrives. The auditory system may benefit (in terms of speech recognition efficiency) in two ways: (a) more resources for speech processing are available, since less or no speech-oscillation synchronism is needed and/or (b) the temporal grid may be more precise, in terms of signal—oscillation synchronicity, compared to the one that would be produced by the word alone. Children in our study may benefit from both, since their Central Auditory Nervous System is still under development (Eggermont, 2014). It should be noted however, that the Speech Segmentation Model does not explain the difference of the rhythm effect's magnitude that was observed between the 1st and 2nd syllable. Results of this study add to the Greek language research on rhythm and are in agreement with a similar rhythmic priming across languages suggesting a domain-general rhythm perceptual mechanism.

### The role of the striato-thalamo-cortical system

A series of studies have demonstrated the implication of the striato-thalamo-cortical system (STCS; comprising the putamen, caudate nucleus, thalamus, supplementary motor area, dorsal premotor cortex, and dorsolateral prefrontal cortex) in perception of rhythmic sequences (Artieda et al., 1992; Pastor et al., 1992; Harrington et al., 1998; Grahn, 2009; Grube et al., 2010; Teki et al., 2011, 2012). It is expected that isochronous beat sequences in this study did activate this system. As enhancement in word recognition was observed due to the rhythm, the authors suggest that STCS may have played a role in the mechanism underlying the effect. It is further proposed that beyond the specific paradigm of this study STCS involvement may play a role in speech segmentation in general.

## Conclusions

This study offers evidence for a positive effect of rhythm stimulation that is synchronized with speech, on speech processing at a lower level than previously reported, i.e., word recognition, and at pre linguistic levels of processing. The authors thus propose that this effect concerns sensory/phonemic processing enhancement, rather than language processing. These findings highlight further the correlations between speech and music/rhythm perception, and the common underpinning mechanisms and pathways that these share. Attention may also be an important factor, driven here by the incoming auditory beat sequences' rhythm. Future research on children with Auditory Processing Disorder is required, in order to investigate the effect of rhythm on speech in noise recognition, possible correlation with other aspects of auditory processing and use of rhythm training to enhance auditory processing abilities.

## Author contributions

CS has tested all individuals in this study, has run the statistics and wrote the paper. VI, IN, and DB have set up the experiment based on an idea by CS and have contributed to the writing of the paper through extensive editing. TR has contributed through editing of the draft as well as through following-up on the idea of the experiment.

### Conflict of interest statement

The authors declare that the research was conducted in the absence of any commercial or financial relationships that could be construed as a potential conflict of interest.

## Footnotes

^1^We thank the anonymous reviewer for pointing this out.

## Abbreviations

WRRC: Word Recognition - Rhythm Component
IBI: Inter Beat Interval
IPI: Inter P-centers Interval
RH: Rhythm Condition
NR: Non-Rhythm Condition
UnSc: Unsynchronized Condition
DAT: Dynamic Attention Theory
STCS: Striato-thalamo-cortical system.
